# A Novel Allotriploid Hybrid Derived From Female Goldfish × Male Bleeker’s Yellow Tail

**DOI:** 10.3389/fgene.2022.880591

**Published:** 2022-04-19

**Authors:** Jing Wang, Weiguo He, Wen Wang, Ziye Luo, Linmei Han, Caixia Xiang, Mingli Chai, Tangluo Li, Jihong Li, Kaikun Luo, Rurong Zhao, Shaojun Liu

**Affiliations:** ^1^ State Key Laboratory of Developmental Biology of Freshwater Fish, College of Life Sciences, Hunan Normal University, Changsha, China; ^2^ Clinical Anatomy and Reproductive Medicine Application Institute, Hengyang Medical School, University of South China, Hengyang, China; ^3^ Hunan Province Cooperative Innovation Center for Molecular Target New Drug Study, Institute of Pharmacy and Pharmacology, University of South China, Hengyang, China

**Keywords:** distant hybridization, allotriploid, inheritance, recombination, sterility

## Abstract

Hybridization is a traditional and effective strategy to alter the genotypes and phenotypes of the offspring, and distant hybridization is a useful strategy to generate polyploids in fish. In this study, goldfish (*Carassius auratus*, GF, 2n = 100) and Bleeker’s yellow tail (*Xenocypris davidi* Bleeker, YT, 2n = 48), which belong to different subfamilies, were crossed with each other. The cross of female GF × male YT successfully obtained hybrid offspring (GFYT hybrids), while the cross of female YT × male GF was lethal, and all the fertilized eggs stopped developing before the neurula stage of embryogenesis. All GFYT hybrids possessed 124 chromosomes (3n = 124) with two sets from GF and one set from YT. The measurable and countable traits of GFYT hybrids were identified, and the genetic characteristics of 5S rDNA between GFYT hybrids and their parents were also revealed. There were, respectively, four and three different 5S rDNA types in GF (assigned as GF-Ⅰ∼Ⅳ) and YT (assigned as YT-Ⅰ∼Ⅲ), and GFYT hybrids specifically inherited YT-Ⅰ and YT-Ⅱ 5S rDNA types from YT and GF-Ⅲ and GF-Ⅳ from GF. In addition, there were only testis-like and fat-like gonads been found in GFYT hybrids. Interestingly, there were pyknotic and heteromorphous chromatin and invaginated cell membrane observed in the spermatids of testis-like gonads, but no mature sperm were found. Furthermore, TUNEL assays indicated that, compared with control, apparent apoptotic signals, which were mainly distributed around spermatid regions, were detected in the testis-like gonads, and the expression of apoptosis pathway-related genes including *p53*, *bcl-2*, *bax*, and *caspase9* was significantly upregulated. Moreover, the expression of meiosis-related genes including *spo11*, *dmc1*, and *rad51* showed an abnormally high expression, but *mns1* and *meig1*, two key genes involved in the maturation of spermatid, were extremely downregulated. In brief, this is the first report of allotriploid *via* distant hybridization between GF and YT that possessing different chromosome numbers in vertebrates. The obtainment of GFYT hybrids not only harbors potential benefits and application in aquaculture but also further extends the understanding of the influence of hybridization and polyploidization on the genomic constitution of the hybrid offspring. Furthermore, they can be used as a model to test the origin and consequences of polyploidization and served as a proper resource to study the underlying mechanisms of spermatogenesis dysfunctions.

## Introduction

Hybridization, including intraspecific hybridization (crossing within a species as a line or strain) and distant hybridization (interspecific or above-specific hybridization) (Bartley et al., 2001; Liu et al., 2007), is the mating of genetically differentiated individuals or groups. It is a useful strategy to alter the genotypes and phenotypes of the offspring *via* transferring the genome of one species to another. Hybridization is thought to facilitate adaptive radiation and speciation in animals (Mallet, 2007; Abbott et al., 2013). By hybridization, the hybrid offspring may inherit the advantageous traits from two parental species, forming a new species with novel heterosis (Liu, 2010), exhibiting advantages in growth rate, disease resistance, viability, fecundity, yield, and appearance (Birchler et al., 2006; Hu et al., 2012). Compared with intraspecific hybridization, distant hybridization is apt to generate heterosis (Chen et al., 2018). In nature, hybridization exists widely in plants and animals, and it is an impetus for speciation and biological evolution (Barton and Hewitt, 1989). Numerous species have hybrid ancestry, presumably due to hybridization between closely related species (Schumer et al., 2016).

Polyploidization, the duplication of the whole genome, is an important driving force for the emergence of evolutionary novelties (Soltis and Soltis, 2009; Zhao et al., 2018). Hybridization, especially distant hybridization, plays vital roles in triggering polyploidization (Mallet, 2007). Polyploidization is an important and frequent event in lower vertebrate evolution, especially that of fish (Venkatesh, 2003), from the sharks to the higher teleosts (Leggatt and Iwama, 2003; Liu et al., 2007; Wang et al., 2019; Wang et al., 2021). In agriculture, hybridization and polyploidization are important breeding technologies. Many artificially induced polyploid fish have been used in aquaculture to produce sterility and to improve production (Liu et al., 2001; Liu et al., 2007). Furthermore, these polyploids are useful model systems to verify the theories about the origin and consequences of polyploidization (Comber and Smith, 2004).

Then, choosing the right or suitable parents and screening the hybrid offspring with improved characteristics becomes one of the main directions of fish genetic breeding (Wang et al., 2015). Goldfish (*Carassius auratus* Var., 2n = 100, GF), within the subfamily of Cyprinidae, genus *Carassius* which are commonly speculated as ancient polyploids (Braasch and Postlethwait, 2012; Knytl and Fornaini, 2021), presents the advantages of a colorful body and strong disease resistance. Bleeker’s yellow tail (*Xenocypris davidi* Bleeker, 2n = 48, YT), within the subfamily of *Xenocyprininae*, possesses the benefit of excellent meat quality. Based on some general rules regarding parental selection for the distant hybridization of fish that were revealed in our previous works (Liu, 2010; Wang et al., 2019), the reciprocal crosses between GF and YT were conducted in this study.

The 5S rDNA multigene family, composed of highly conserved 5S rRNA sequence and variable non-transcribed spacer (NTS) which form arrays of hundreds to thousands of tandem repeats, presents species-specific characteristics in length and the composition of nucleotide sequence (He et al., 2012; He et al., 2013). The accumulation of results from fish indicates 5S rDNA as a valuable marker for identification (Martins and Galetti, 2001; Wasko et al., 2001; Pasolini et al., 2006), particularly in hybrids (Qin et al., 2010; He et al., 2012; He et al., 2013).

In addition to the embryonic development, the ploidy (DNA content and chromosome composition), and the morphological characteristics (countable and measurable traits), the heredity of 5S rDNA and the gonadal development of the hybrids were also observed and assayed. Briefly, this is the first report of allotriploid *via* distant hybridization between GF and YT that possessing different chromosome numbers in vertebrates. The obtainment of GFYT hybrids has potential benefits and application in aquaculture. In addition, the variation analysis of 5S rDNA multigene families further extended the understanding of the influence of hybridization and polyploidization on the genomic constitution of the hybrid offspring. Furthermore, they can be used as a model to test the origin and consequences of polyploidization. Moreover, this novel allotriploid, which harbors only testis-like and fat-like gonads, can also serve as a proper resource to study the underlying mechanisms of spermatogenesis dysfunctions.

## Materials and Methods

### Animals

All fish, including GF, YT, and GFYT hybrids, were cultured in ponds (23–27°C, natural light cycle) at the State Key Laboratory of Developmental Biology of Freshwater Fish and artificially fed. All experiments were approved by the Animal Care Committee of Hunan Normal University and followed the guidelines of the Administration of Affairs Concerning Animal Experimentation of China.

### Obtainment of GFYT Hybrids

During the breeding seasons (April to June), the crosses of GF (2n = 100, ♀, n = 3) × YT (2n = 48, ♂, n = 3) and YT (2n = 48, ♀, n = 3) × GF (2n = 100, ♂, n = 3) were conducted, respectively. Two self-crossings of GF and YT (n = 6 each, with the same sex ratio) were also performed. The zygotes were cultured in dishes at a water temperature of 19–20°C. About 2,000 embryos were chosen at random to measure the fertilization rate (number of embryos at the gastrula stage/number of eggs × 100%) and hatching rate (number of hatched fry/number of eggs × 100%). The main stages of embryogenesis were photographed using a stereoscope (Leica, MZ16FA, Wetzlar, Germany). Finally, the hatched fry was transferred to different ponds for further culture.

### Measurement of Measurable and Countable Traits

For measurable traits, one-year-old GF, YT, and GFYT hybrids (230–350 g, n = 30 each, including parents) were randomly selected to check total length (TL), body length (BL), body height (BH), head length (HL), head height (HH), caudal peduncle length (CPL), and caudal peduncle height (CPH) (with 0.1cm accuracy). Then, the ratios of BH/BL, BL/TL, HL/BL, HH/HL, CPH/CPL, and HH/BH were further calculated. In addition, the number of lateral line scales (LS), scale rows above the lateral line (ALS), scale rows below the lateral line (BLS), dorsal fin rays (DFR), anal fin rays (AFR), and pelvic fin rays (PFR) were also counted.

### Flow Cytometry

The peripheral blood, taken from the caudal veins of 5 GF, 5 YT, and 30 GFYT hybrids which were older than 6 months, were filtered and stained with 4’, 6-Diamidino-2-phenylindole DNA-staining solution, cystain DNA 1 step (Partec, Görlitz, Germany). The mean DNA content of GF, YT, and GFYT hybrids was measured with flow cytometry (Ploidy Analyzer PA, Partec, Münster, Germany). Finally, a comparison of mean DNA content between GFYT hybrids and their parents was tested by the χ^2^ test with SPSS (version 17.0, the Chi-square test with Yates correction was used for testing deviation from the expected ratio values).

### Chromosome Spreads

The preparation of metaphase chromosome spreads was performed with the kidney tissues and the protocols were carried out according to Wang et al. (2014).

### Fluorescence *In Situ* Hybridization

A specific probe of 263 bp centromere repeat sequence, amplified from the genomic DNA of GF, and labeled with Dig-11-dUTP via using a PCR DIG probe synthesis kit (Roche, Penzberg, Germany), was hybridized with the metaphase chromosome spreads of GFYT hybrids and their parents (n = 3 each) according to the previous method described by He et al. (2012). The primer pair (5′-AAG​CTT​TTC​TCT​CTA​GTA​GAG​AAA​GC-3′; 5′-TTG​AGC​AGA​TTT​GGG​CTT​GAT​TTC-3′, sequence number: JQ086761) was used to amplify this specific probe and at least 30 metaphases from each sample were analyzed.

### Histological Analysis of Gonadal Tissues

Compared with one-year-old female GF and male YT, 20 GFYT hybrids at the same age were randomly selected to check the histological structure of gonads. First, the gonadal tissues were fixed in Bouin’s solution overnight. The paraffin sections (6 μM) were sequentially cut, deparaffined, and further stained with hematoxylin and eosin. The microstructure of gonadal tissues was photographed and performed under a light microscope (Olympus, CKX41-32PH, Tokyo, Japan). Meantime, some other gonadal tissues were sequentially fixed in 3% glutaraldehyde solution, washed with phosphate buffer, transferred into osmic acid solution, dehydrated in a graded acetone series, and finally embedded in Epon 812. Ultrathin sections (60 nM) were cut and stained with uranyl acetate and lead citrate. The ultrastructure of gonadal tissues was observed and photographed with an electron microscope (HITACHI, HT7800, Tokyo, Japan).

### Terminal Deoxynucleotidyl Transferase dUTP Nick End-Labeling Assay

Gonadal tissues from one-year-old testis-like GFYT hybrids and male GF (n = 3 each) were embedded in the optimal cutting temperature compound (SAKURA, Torrance, CA, United States). The apoptotic analysis was performed on sections (6 mm) by using the *in situ* cell death detection kit (Roche, Penzberg, Germany) according to the manufacturer’s instructions. Apoptotic signals were observed using a microscope (Leica, DM6000B, Wetzlar, Germany).

### 5S rDNA Assay

Total genomic DNA was, respectively, isolated from the blood cells of GF, YT, and GFYT hybrids with a DNA extraction kit by following the manufacturer’s instructions (Sangon, Shanghai, China). The reaction mixture (25 μl) consisted of 20 ng genomic DNA, 1.5 mM MgCl_2_, 0.2 mM of each dNTP, 0.4 μM of each primer, 1 × PCR buffer, and 1.25 U Taq polymerase (Takara, Dalian, China). The amplification process started with an initial denaturation at 94°C for 4 min, then followed by 30 cycles of 94°C for 30 s, 60°C for 30 s, and 72°C for 1 min. A final extension step was performed at 72°C for 10 min. The PCR products were sequentially separated on a 1.2% agarose gel, purified using a gel extraction kit (Sangon, Shanghai, China), ligated into a pMD18-T vector, and transferred into *E. coli* DH5α. Finally, the positive clones were then sequenced using an automated DNA sequencer (ABI PRISM 3730: Applied Biosystems, Carlsbad, CA, United States). Ten clones of each PCR band were sequenced. The primers used for 5S rDNA amplification were listed in Table 1.

**TABLE 1:** List of primers used in this study.

Gene	Sequence(5'→3′)	Annealing temperature (°C)	Primer purpose
5s rDNA	F: GCT​ATG​CCC​GAT​CTC​GTC​TGAR: CAG​GTT​GGT​ATG​GCC​GTA​AGC	60	PCR
*dmc1*	F: GAA​GAT​GAG​GAA​TCC​TTT​TTT​CA	56	qPCR
	R: GCC​TCA​GAC​AGT​CCC​TTT​ACA​TT		
*spo11*	F: CAG​TTT​CCT​CAC​CAT​CAG​TCT​GC	55	
	R: CTT​CCA​ATG​TTA​ATG​GGA​TCA​GA		
*rad51*	F: AGT​GGA​GGA​AGA​GGA​GAA​TT	56	
	R: TCT​CAC​CTC​TGC​CTT​TCC​T		
*mns1*	F: CCA​ACC​CTC​ATC​AGA​GAA​AAT​AT	55	
	R: GAT​CAT​TGA​AGA​GGA​GAG​ACA​GA		
*meig1*	F: GTC​GAG​GAG​CTG​TCA​GTA​CAC​AT	60	
	R: ACA​GAG​ATG​AGC​TGG​AGT​ACC​GA		
*p53*	F: CCC​ATC​CTC​ACA​ATC​ATC​ACT​C	56	
	R: TCT​TGC​TTG​GGG​TTT​TGG​TCT​C		
*bcl-2*	F: CAG​TAT​AGT​GGT​GAA​GTA​CAT​CC	55	
	R: TCT​CCA​TCA​TCA​GTC​CAT​TCG		
*bax*	F: ATT​TCC​TGT​CTC​TTC​CTC​ATC​C	55	
	R: AGA​ACA​CGA​GTG​CTG​ATT​GG		
*Caspase9*	F: GAT​GGA​GCC​CTC​TTT​TGG​TGG	60	
	R: GAG​ACG​TAG​CCT​GGA​AAG​GT		
*β*-actin	F: GCC​CTG​CCC​CAT​GCC​ATC​CT	62	
	R: AGT​GCC​CAT​CTC​CTG​CTC​GA		

### Real-Time Quantitative PCR

Total RNA was extracted from the gonads of male GF and testis-like GFYT hybrids (n = 3 each) with TRIzol™ Reagent (Invitrogen, Carlsbad, CA, United States). The concentration and purity of RNA were determined using spectrophotometry and agarose gel electrophoresis. After treatment with RNase-free DNase (Promega, Madison, WI, United States), the total RNA was reverse transcribed to complementary DNA (cDNA) with Rever Tra Ace M-MLV (TOYOBO, Osaka, Japan). RT-qPCR was carried out by using 23SYBR Green mix (TOYOBO, Osaka, Japan) and ABI 7900HT RT-PCR System (Applied Biosystems, Carlsbad, CA, United States). *β-actin* was used as the internal reference and the expression levels were calculated with the 2^−ΔΔCt^ method. The primers used here were listed in Table 1.

### Statistical Analysis

All statistical analyses were conducted by using SPSS software (version 17.0). Differences between multiple groups were analyzed by one-way analysis of variance (ANOVA) (IBM Corp. New York, United States). Sigma Plot 10.0 (San Jose, CA, United States) was used to present the basic graph. All data were shown as means ± SD. A *p*-value less than 0.05 was considered to be statistically significant.

## Results

### The Formation of GFYT Hybrids and Their Embryonic Development

**FIGURE 1:**
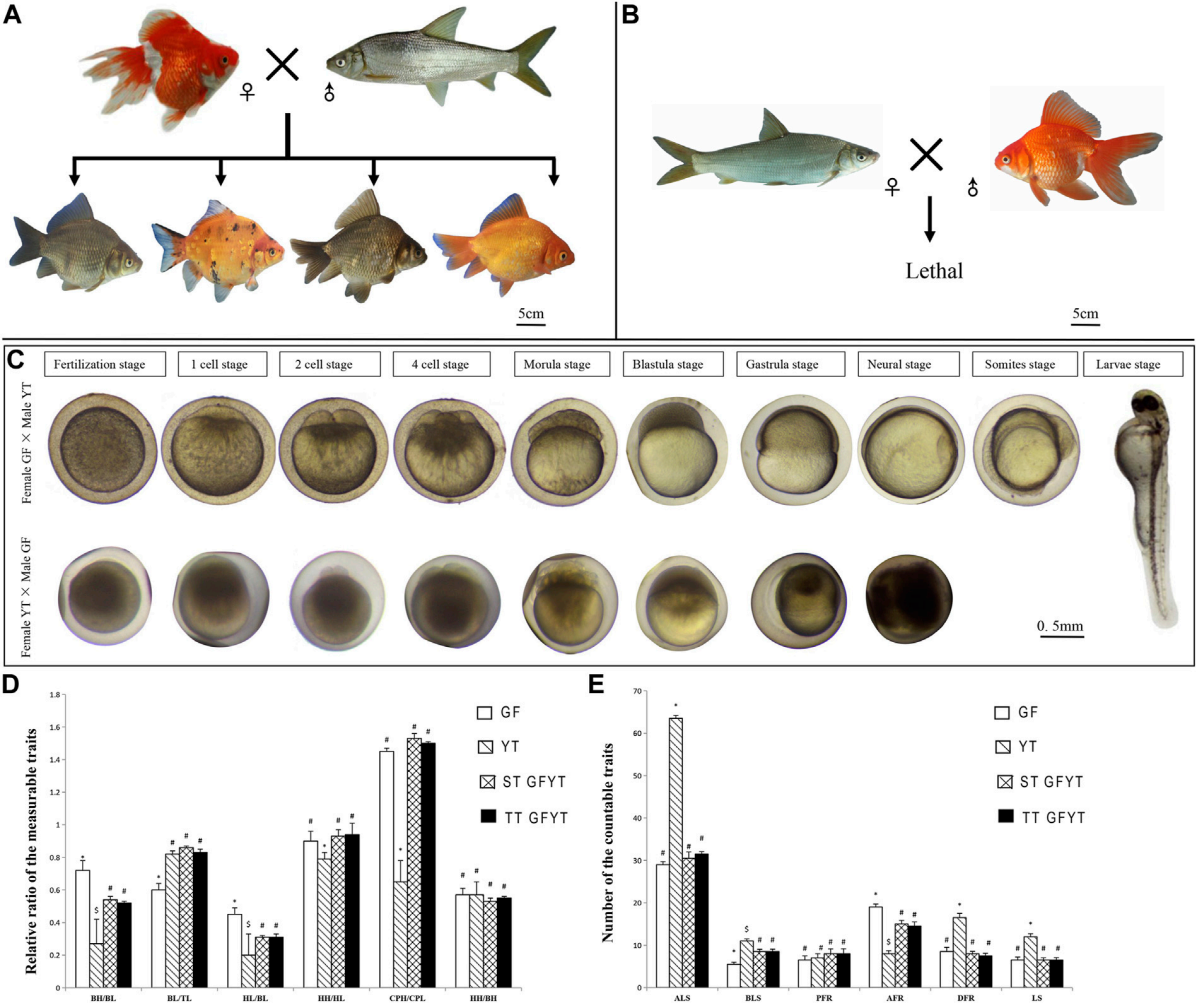
Reciprocal crosses, main stages of embryonic development, and comparisons of morphological traits between GFYT hybrids and their parents. **(A)** Cross of female GF × male YT and their hybrid offspring. **(B)** Lethal cross of female YT × male GF. **(C)** Main stages of embryonic development of reciprocal crosses. The upper lane shows the cross of female GF × male YT, and the lower presents the cross of female YT × male GF. **(D)** Comparisons of the measurable traits between GFYT and their parents. **(E)** Comparisons of the countable traits between GFYT hybrids and their parents. For each comparison, different symbols (# or *) mean significant difference (*p* < 0.05). TL, total length; BL, body length; BH, body height; HL, head length; HH, head height; CPL, caudal peduncle length; CPH, caudal peduncle height; LS, lateral line scales; ALS, scale rows above the lateral line; BLS, scale rows below the lateral line; DFR, dorsal fin rays; PFR, pelvic fin rays; AFR, anal fin rays; ST, single tail; TT, twin tail.

The crosses of GF (♀) × YT (♂) in which the number of maternal chromosomes is more than that of paternal chromosomes generated four types of hybrid offspring, including gray or red single-tail individuals (ST GFYT hybrids) and gray or red twin-tail individuals (TT GFYT hybrids) (Figure 1A). While the crosses of YT (♀) × GF (♂) in which the number of maternal chromosomes is less than that of paternal chromosomes were lethal (Figure 1B). In addition, embryogenesis was also observed. Most of the embryos from the cross of GF (♀) × YT (♂) showed normal development, but all the embryos from the cross of YT (♀) × GF (♂) died before the end of the neurula stage (Figure 1C). Meantime, the fertilization rates and hatching rates were further analyzed (Table 2). There were high fertilization rates and hatching rates in both the self-crossing of GF (94.3 and 86.7%, respectively) and YT (90.2 and 83.4%, respectively). Also, the fertilization rates and hatching rates of GF (♀) × YT (♂) were 75.3 and 64.6%, respectively. However, the fertilization rates and hatching rates of YT (♀) × GF (♂) were severely reduced to 5.0% and 0.

**TABLE 2:** Fertilization rates and hatching rates of different crosses.

Crossing group	Fertilization rates	Hatching rates
GF(♀)× GF(♂)	94.3%	86.7%
YT(♀)× YT(♂)	90.2%	83.4%
GF(♀)× YT(♂)	75.3%	64.6%
YT(♀)× GF(♂)	5.0%	0

### Measurable and Countable Traits of GFYT Hybrids

The comparisons of measurable and countable traits among GFYT hybrids and their parents were also conducted (Figures 1D,E). There was no significant difference between single-tail and twin-tail individuals. Compared with parents GF and YT, the rates of the main characteristics, including body height/body length and head length/body length of GFYT hybrids presented obvious intermediate properties. But the rate of body length/total length of GFYT hybrids showed an apparent bias to that of YT, and the rates of head height/head length and caudal peduncle height/caudal peduncle length presented higher similarity to that of GF. The countable traits of GFYT hybrids were also more similar to that of GF, excepting BLS.

### DNA Content and Chromosomal Constitution of GFYT Hybrids

**FIGURE 2:**
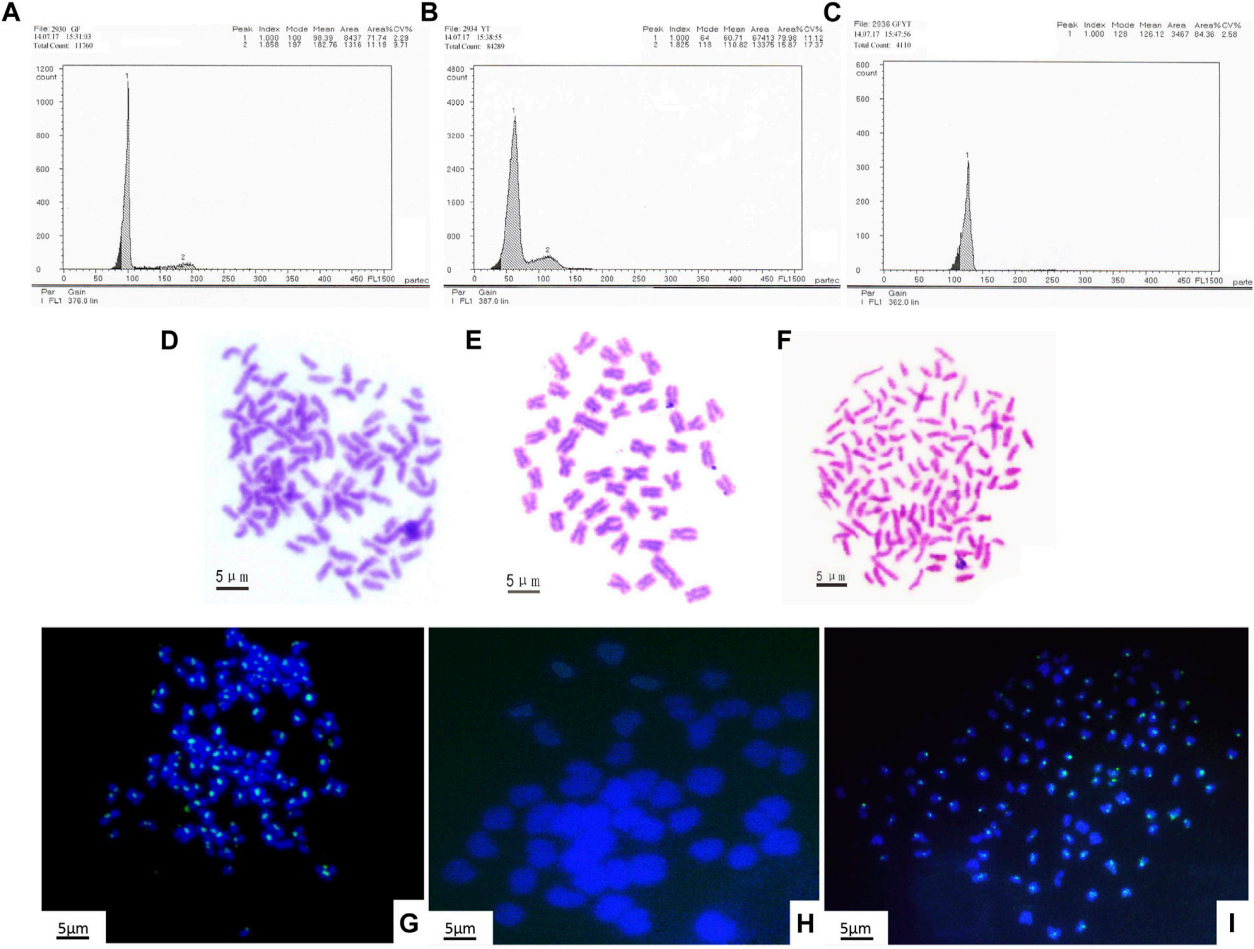
Flow cytometric histograms and chromosomal constitution of GFYT hybrids and their parents. **(A–C)** Mean DNA content. **(A)** GF (peak 1: 98.39). **(B)** YT (peak 1: 60.71). **(C)** GFYT hybrids (peak 1: 126.12). **(D–F)** Chromosome spreads at metaphase. **(D)** 100 chromosomes of GF. **(E)** 48 chromosomes of YT. **(F)** 124 chromosomes of GFYT hybrids. **(G–I)** Fluorescence *in situ* hybridization of mitotic metaphase chromosomes. **(G)** GF, about 100 signals. **(H)** YT, no signal. **(I)** GFYT, about 100 signals.

To reveal the construction of genetic materials (nucleus), the mean DNA contents of GFYT hybrids and their parents were checked. There was no significant difference between the value of GFYT/(GF + 0.5YT) and the expected value (Figures 2 A–C, Table 3). Then, we further confirmed the chromosomal constitution of GFYT hybrids and their parents. There were 100 and 48 chromosomes in GF and YT, respectively. All GFYT hybrids possessed 124 chromosomes which was equal to the number of GF chromosomes and one-half of YT chromosomes (Figures 2D–F). To further confirm the chromosomal heredity of GFYT hybrids, a specific probe from GF was applied to hybridize the metaphase chromosome spreads of GFYT hybrids and their parents. There were 100 signals in GF and none in YT. As expected, only 100 signals were found in the GFYT hybrids (Figures 2G–I). Then, a novel allotriploid population with 124 chromosomes, with two sets from GF and one set from YT, was successfully obtained from the cross of GF (♀) × YT (♂).

**TABLE 3:** Mean DNA content in GF, YT, and GFYT.

Fish type	Mean DNA content	Specific value	
		Observed value	Expected value
GF	98.39		
YT	60.71		
GFYT	126.12	GFYT/(GF + 0.5YT) = 0.98^a^	1

a The observed ratio was not significantly different (p > 0.01) from the expected ratio.

### Heredity of 5S rDNA in GFYT Hybrids

**FIGURE 3:**
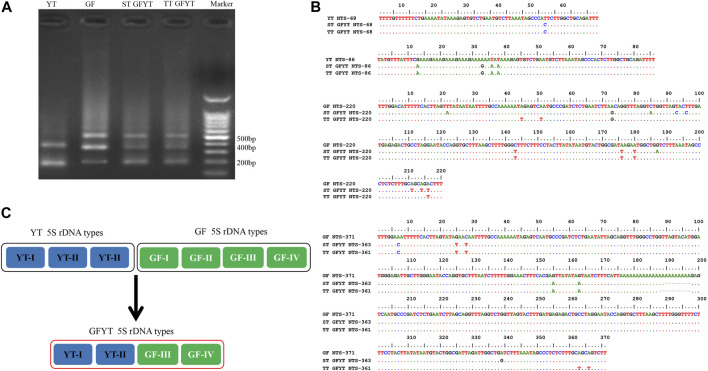
Hereditary characteristics of 5S rDNA units in GFYT hybrids and their parents. **(A)** DNA bands amplified from GF, YT, and GFYT hybrids. Marker: DNA ladder markers with 100 bp increments. **(B)** Alignment of the NTS sequences. **(C)** Ideogram presenting the genetic constitution of 5S rDNA units in GFYT hybrids.

To reveal the characteristics of molecular heredity of GFYT hybrids, the composition, and organization of 5S rDNA in GFYT hybrids and their parents was checked. There were distinctive band patterns on the agarose gel electrophoresis, including two (approximately 200 and 400 bp) in YT and three (approximately 200, 400, and 500 bp) in GF and GFYT hybrids (Figure 3A). The sequencing results indicated that there were three different sizes (188, 206, and 376 bp) in YT, four (168, 203, 340, and 483 bp) in GF, four (188, 206, 340, and 484 bp) in ST GFYT hybrids and four (188, 206, 340, and 482 bp) in TT GFYT hybrids (Table 4), respectively. Based on BLASTn analysis (https://blast.ncbi.nlm.nih.gov/Blast), all fragments were confirmed to be 5S rDNA repeat units. According to the length, the similarities of 5S rDNA repeat units between GF and YT were first checked. There were extremely low similarities between GF-168 and YT-188 (72.8%), GF-203 and YT-206 (76.5%), and GF-340 and YT-376 (70.8%). So, the 5S rDNA composition of GF and YT were, respectively, assigned as GF-Ⅰ∼Ⅳ and YT-Ⅰ∼Ⅲ (Table 4). Then, the sequence alignment between GFYT hybrids and their parents was conducted. The 188 bp and 206 bp 5S rDNA of GFYT hybrids (including ST and TT), respectively, showed a high similarity to that of YT-Ⅰ and YT-Ⅱ but obviously low to that of GF-Ⅰ and GF-Ⅱ (values in the brackets). Inversely, the 340 bp 5S rDNA of GFYT hybrids presented a high similarity to that of GF-Ⅲ, but apparently low to that of YT-Ⅲ (value in the brackets). The ∼480 bp 5S rDNA of GFYT hybrids exhibited high similarity to that of GF-Ⅳ (Table 5). In addition, the 5S rDNA sequences with the same or similar length between ST and TT GFYT hybrids showed high similarity (Table 5). Given the high conservation of coding region and variability of NTS in 5S rDNA sequences, the alignment of NTS sequences with the same length between GFYT hybrids and parents was further conducted to confirm their composition, which presented high consistence except several nucleotide mutations (Figure 3B). These results indicated that GFYT hybrids, including single-tail and double-tail individuals, both specifically inherited YT-Ⅰ and YT-Ⅱ 5S rDNA types from YT, GF-Ⅲ, and GF-Ⅳ from GF (Figure 3C).

**TABLE 4:** Results of 5S rDNA sequencing.

Fish type	∼200bp	∼400bp	∼500bp
YT	188bp (YT-Ⅰ), 206bp (YT-Ⅱ)	376bp (YT-Ⅲ)	
GF	168bp (GF-Ⅰ), 203bp (GF-Ⅱ)	340bp (GF-Ⅲ)	483bp (GF-Ⅳ)
ST GFYT	188bp (YT-Ⅰ), 206bp (YT-Ⅱ)	340bp (GF-Ⅲ)	484bp (GF-Ⅳ)
TT GFYT	188bp (YT-Ⅰ), 206bp (YT-Ⅱ)	340bp (GF-Ⅲ)	482bp (GF-Ⅳ)

∼ The approximate size of bands on the agarose gel.

**TABLE 5:** Similarities of 5S rDNA sequences between hybrids and their parents.

Length	ST GFYT/GF	ST GFYT/YT	TT GFYT/GF	TT GFYT/YT	ST GFYT/TT GFYT
188 (168) bp	(72.8%)	96.8%	(75.5%)	97.3%	99.4%
206 (203) bp	(77.4%)	98.1%	(78.4%)	98.1%	100%
340 (376) bp	95.1%	(66.3%)	96.7%	(70.6%)	97.1%
∼480bp	99.5%	-----	99.3%	----	98.9%

∼ The approximate length of 5S rDNA. The numbers or values of percentage in the brackets, respectively, show the base length of 5S rDNA, similar to GF, or YT, and similarities between them and GFYT hybrids.

### Reproduction Dysfunction of GFYT Hybrids

**FIGURE 4:**
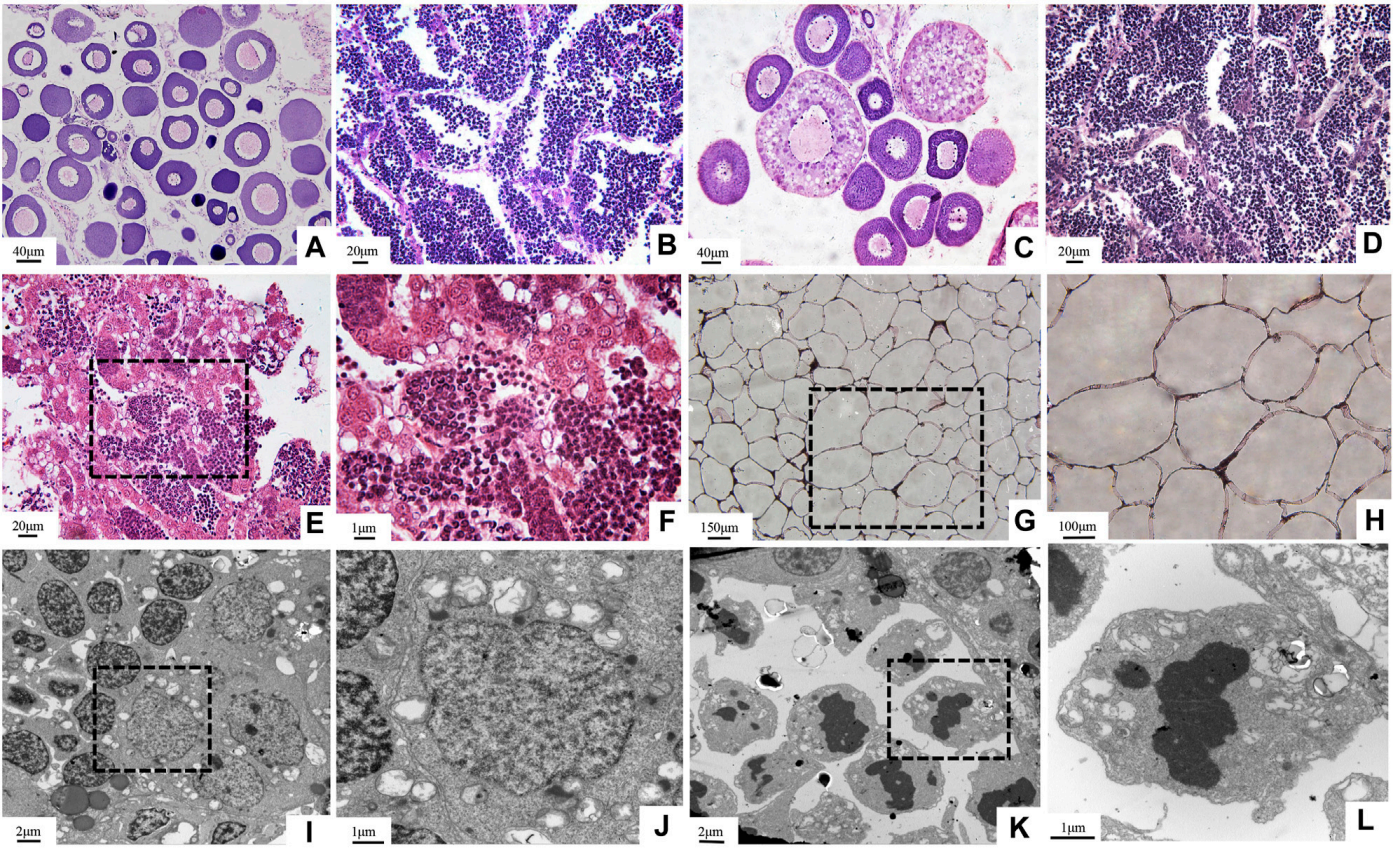
Histological structure of gonads in GFYT hybrids and their parents. **(A–H)** HE-stained gonads and **(I–L)** ultrastructure of testis-like gonads of GFYT hybrids. **(A)** Ovary of GF. **(B)** Testis of GF. **(C)** Ovary of YT. **(D)** Testis of YT. **(E,F)** Testis-like gonad of GFYT hybrids. **(G,H)** Fat-like gonad of GFYT hybrids. **(I,J)** Spermatogonia and spermatocytes in the testis-like gonad of GFYT hybrids. **(K,L)** Spermatids with heteromorphous nuclei in the testis-like gonad of GFYT hybrids. F, H, J, and L, respectively present the amplification of the dashed box in E, G, I, and K.

In the breeding season, compared with the parents, none of the ovum or sperm could be squeezed out from GFYT hybrids. Then, further histological observations were employed to confirm the gonadal development (Figure 4). Both GF (Figures 4A,B) and YT (Figures 4C,D), respectively, present normal oogenesis and spermatogenesis. However, only testis-like (Figures 4E,F) and fat-like gonads (Figures 4G,H) were found in GFYT hybrids. In the testis-like gonads, there were spermatogonia, spermatocytes, and spermatids, while no mature sperm was found (Figures 4E,F). Interestingly, the electron microscope did not observe abnormality in the structure of spermatogonia and spermatocytes (Figures 4I,J) but found that spermatids presented an abnormal organization with pyknotic and heteromorphous chromatin and invaginated cell membrane (Figures 4K,L), indicating the undergoing degeneration.

### Apoptosis of Testis-Like Gonads in GFYT Hybrids

**FIGURE 5:**
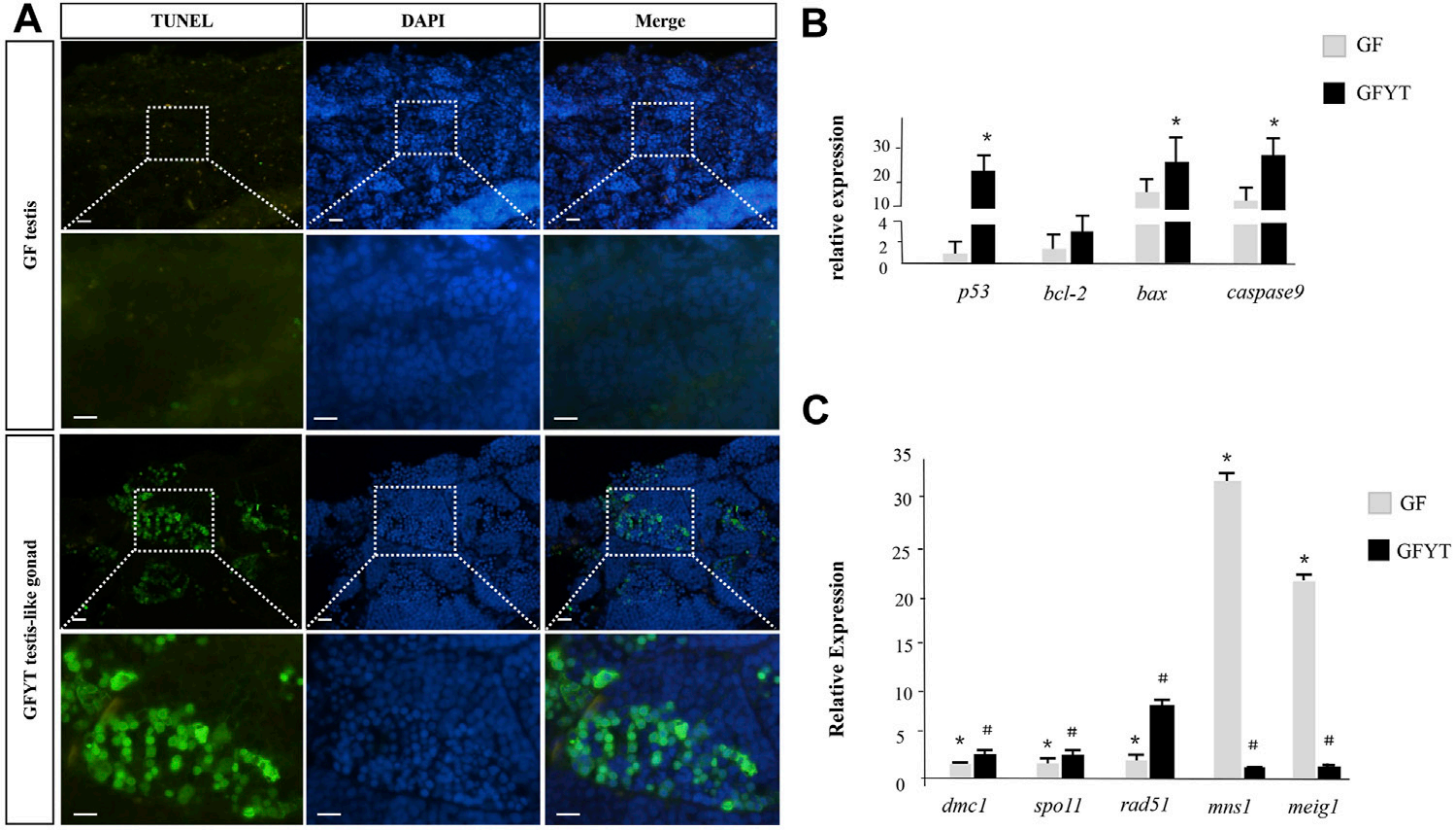
Apoptosis assay and spermatogenesis-related gene expression in the testis of GF and GFYT hybrids. **(A)** TUNEL analysis of the testis of GF and testis-like gonad of GFYT hybrids, Bar = 20 μM. **(B)** Real-time PCR quantification of *p53*, *bcl-2, bax*, and *caspase9*. **(C)** Real-time PCR quantification of *dmc1*, *spo11*, *rad51*, *mns1*, *and meig1*. Columns labeled with different symbols (# or *) mean significant difference (*p* < 0.05).

The newly formed allotriploid were proved to be sterile and the spermatids presented obvious degeneration (Figure 4). These results suggested that the infertility of GFYT hybrids may result from the developmental arrestment of spermatogenesis. Apoptosis, a programmed cell death, which is crucial for the normal development and organismal homeostasis maintenance (Singh et al., 2019), holds the highest possibility. To uncover the underlying mechanisms, the TUNEL assays were conducted. Compared with male GF whose testis showed nearly no signal, apparent apoptotic signals were mainly distributed around spermatid regions in the testis-like gonads of GFYT hybrids (Figure 5A). Then, the mRNA expression of apoptosis pathway-related genes, including *p53*, *bcl-2*, *bax*, and *caspase9* that play vital roles in the normal development of germ cells and the reproductive system (Singh et al., 2019) were further identified (Figure 5B). Compared with control, though there was only an increasing trend in the mRNA expression of *bcl-2*, the expression of *p53*, *bax*, and *caspase9* of testis-like gonads in GFYT hybrids showed an observable upregulation (Figure 5B). In addition, to explore the possible factors causing the prevention of spermatogenesis, the mRNA expression of several key meiosis or spermatid maturation related genes was checked. The expression of meiosis-related genes, including *spo11, dmc1*, and *rad51* showed an abnormality of high expression in the testis-like gonad of GFYT hybrids (Figure 5C). However, the expression of *mns1* and *meig1*, two key genes involving the maturation of spermatid, were extremely downregulated (Figure 5C).

## Discussion

A novel and sterile allotriploid was successfully obtained *via* crossing female GF and male YT, which belonged to different subfamilies in this study. The composition and similarity assays of 5s rDNA were also conducted to reveal the genetic characteristics of GFYT hybrids. In addition, the gonadal dysfunctions and spermatogenesis disorders were further analyzed to explore the underlying mechanisms.

### Novel Alloploid Produced by Hybridization

Distant hybridization is a valuable approach for producing hybrid offspring with improved characteristics in fish breeding. It may break through reproductive barriers, integrate advanced characteristics from different species, expand the genetic variation, or even produce new variants or species (Coyne and Orr, 2004; Mallet 2007). However, most hybridization resulted in hybrid incompatibility (Coyne and Orr, 2004; Mallet, 2005; Maheshwari and Barbash, 2011; Abbott et al., 2013). Many genetic and environmental factors are the cause of hybrid incompatibility. Among them, the genetic factors included conflict in the interaction of different genes, allele variants or epigenome, the conflict between the maternally inherited cytoplasm and the hybrid genome, and conflict coming from the biased expression of either parental alleles in the hybrid offspring (Vaid and Laitinen, 2019). In this study, GF and YT, respectively, possess 100 and 48 chromosomes, only the cross of GF (♀) × YT (♂) in which the number of maternal chromosomes is more than that of paternal chromosomes generated surviving hybrid offspring, but the cross of YT (♀) × GF (♂) in which the number of maternal chromosomes is less than that of paternal chromosomes was lethal. These results were consistent with the previous studies which stated that it is more likely to obtain hybrid offspring when the number of maternal chromosomes is larger than that of paternal chromosomes, but the hybrids are unlikely to survive if the number of maternal chromosomes is fewer than that of paternal chromosomes (Liu, 2010; Song et al., 2012; Wang et al., 2019).

To conquer hybrid incompatibility, the hybrid offspring may rearrange the genome (Osabe et al., 2012). The 5S rDNA multigene family presents species-specific characteristics in length and the composition of the nucleotide sequence. The accumulation of results from fish indicates 5S rDNA could be used to identify species and analyze the evolution and genetic relationship (Sajdak et al., 1998; Campo et al., 2009), especially hybrid progeny (Pendas et al., 1995; Qin et al., 2010; He et al., 2012). Herein, GFYT hybrids including single-tail and twin-tail individuals specifically inherited the paternal-specific YT-Ⅰ and YT-Ⅱ 5S rDNA types, maternal-specific GF-Ⅲ and GF-Ⅳ. However, the paternal-specific YT-Ⅲ, maternal-specific GF-Ⅰ, and GF-Ⅱ 5S rDNA types were lost in the hybrid offspring. In addition, several variations of nucleotide sequence were also found in NTS sequences. These variations of NTS sequences suggested that 5S rDNA can be used as suitable genetic markers for the identification of GFYT hybrids and their parents.

**FIGURE 6:**
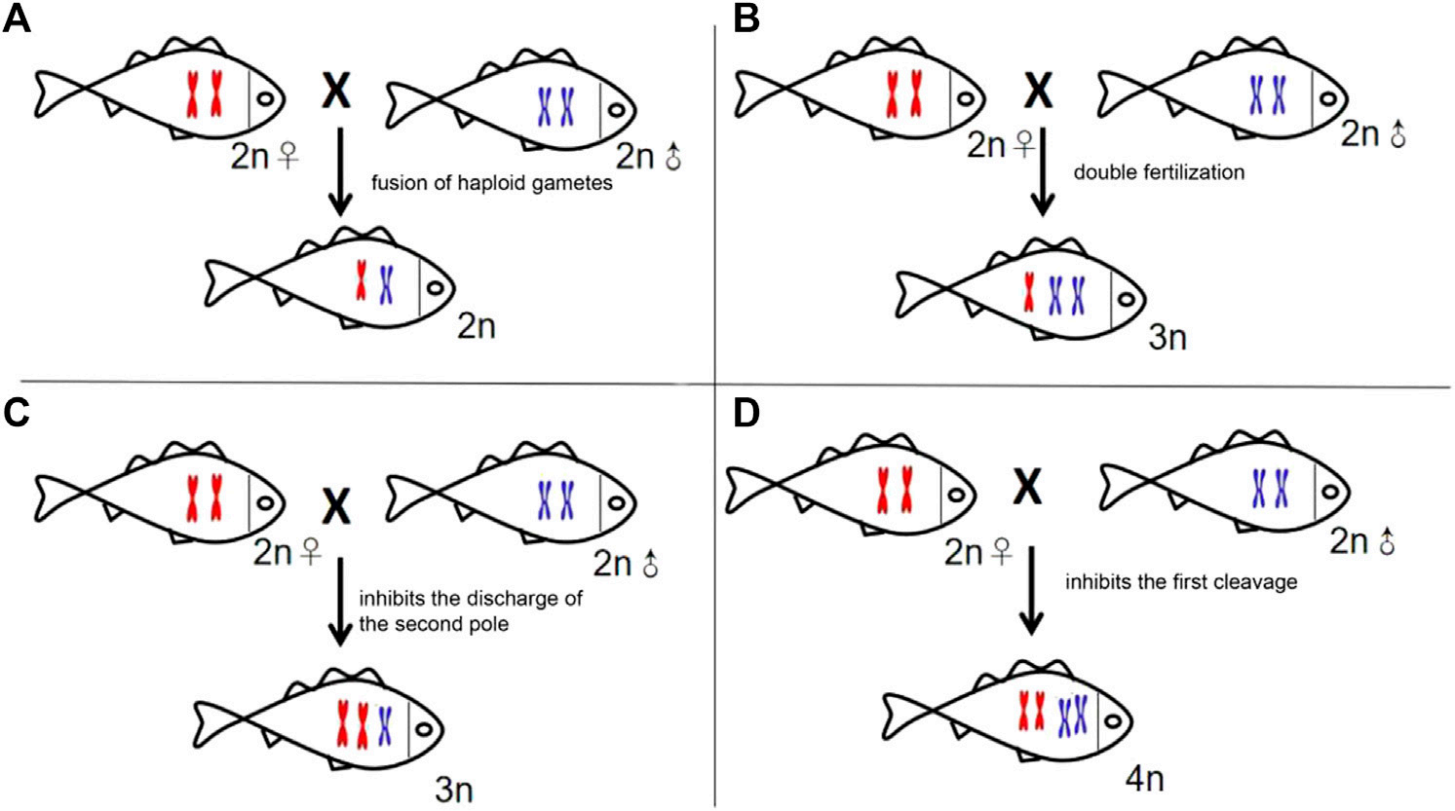
Schematic presenting the four types of formation mechanisms of the heterogeneous hybridization offspring. **(A)** Allodiploid resulting from the fusion of haploid gametes. **(B)** Allotriploid deriving from double fertilization. **(C)** Allotriploid resulting from the expulsion inhibition of the second pole. **(D)** Allotetraploid deriving from the impeding of the first cleavage.

Polyploidization, an important approach to hybrid speciation (Soltis and Soltis, 2009), resulted in a drastic change in the genome mainly through the expulsion inhibition of the second pole, the impeding of first cleavage, or double fertilization (Ramsey and Schemske, 1998). There may be theoretically four types of offspring in the cross of GF (♀) × YT (♂), the allodipliod (2n = 74) with one set of GF and YT, respectively; allotriploid (3n = 98) derived from the double fertilization between one egg from GF and two sperms from YT; allotetraploid (4n = 148) resulting from the obstruction of the first cleavage, and allotriploid (3n = 124) from the inhibition of the expulsion of the second pole (Figure 6). Given the fact that GFYT hybrids were all triploids with two sets of maternal chromosomes and one set of paternal chromosomes, we discreetly concluded that the GFYT hybrids resulted from the inhibition of the expulsion of the second pole. The remaining three types of chromosomal constitution may also exist, but these genetic architectures are lethal during early embryogenesis. So, this is why there were low fertility rates (75.3%) and survival rates (64.6%) in GFYT hybrids.

In brief, this is the first report of allotriploid via distant hybridization between GF and YT which possess different chromosome numbers in vertebrates. The obtainment of GFYT hybrids has potential benefits and applications in aquaculture. In addition, the variation analysis of 5S rDNA multigene families further extended the understanding of the influence of hybridization and polyploidization on the genomic constitution of the hybrid offspring. Furthermore, they can be used as a model to test the origin and consequences of polyploidization.

### Hybrid Sterility

The complex processes involving the pairing, synapsis, recombination, and the separation of homologous chromosomes would sequentially occur in meiotic prophase I to form secondary meiocytes (Pawlowski and Cande, 2005). In this study, there were two sets of chromosomes from GF and one set of chromosomes from YT in GFYT hybrids, which resulted in the failed synaptonemal complex (SC) and irregular division in meiotic prophase I. Then, the sister chromosomes separated and formed aneuploidy spermatids. So, spermatids with pyknotic and heteromorphous chromatin and invaginated cell membrane were found in the testis-like gonads of GFYT hybrids. In addition, apoptotic signals that are mainly distributed around spermatid regions further confirmed this speculation. Furthermore, the BCL-2 family of proteins, whose homeostasis of anti-apoptotic and pro-apoptotic compositions determines the final fate of cells (Deng et al., 2007; Chonghaile et al., 2011; Sarosiek et al., 2013; Sarosiek and Letai, 2016; Singh et al., 2019), are the key regulators of the intrinsic apoptosis (Fuchs and Steller, 2011; Galluzzi et al., 2018). Pro-apoptotic protein Bax activates the caspase signaling pathway to initiate apoptosis, and the anti-apoptotic protein BCL-2 suppresses Bax to prevent apoptosis (Agca et al., 2012; Liman et al., 2013; Lucinda et al., 2013). In this study, the expression of *p53*, *bax*, and *caspase9* significantly increased; however, there was only an increasing trend of *bcl-2* without significance. Interestingly, the meiosis-related genes (*spo11*, *dmc1*, and *rad51*) presented an obvious abnormality of high expression, while *mns1* and *meig1*, two key genes involving the maturation of spermatid, showed extreme downregulation. Why there was a high expression of these meiosis-related genes? One potential explanation is compared with male GF, who finished meiosis at the reproduction season, delayed spermatocytes in the testis-like gonads of GFYT hybrids were still undergoing meiosis, even though this was abnormal. So, these meiosis-related genes were transcribed persistently. These results suggested that heterology and triploidy were more likely the reasons for the sterility of GFYT hybrids.

In addition, the conflicts between the growing demand for aquatic food consumption and the global decline of wild fishery stocks make it imperative to develop highly efficient aquaculture practices to increase fishery production. Then, farming infertile fish is a desirable and most effective genetic-containment strategy for aquatic food availability and the development of environmentally responsible aquaculture (Wong and Zohar, 2015). First, there are close linkages between growth and reproduction of fish (Hill, 2013). Sterilization not only increases the growth of muscle by reducing energy input to gonad development but also inhibits sexual maturation, which would deteriorate flesh quality and enhance susceptibility to disease and stress (Zohar, 1989). Second, the escape of non-native selectively bred or genetically modified (transgenic) fish from aquatic farms would propagate or interbreed with wild stock, thus finally greatly threatening the ecosystem and environment. The sterile trait could significantly alleviate these risks (Muir and Howard, 1999). Third, there are different processes, such as commonly used chromosome manipulation, interspecies hybridization, or newly invented bath-immersion technology (Wong and Zohar, 2015), to induce sterile fish. However, these manipulations would be time-consuming and/or could not be produced on a big scale or the hybrid offspring is fertile. GFYT hybrids, obtained via direct distant hybridization, are all allotriploid and completely sterile, well conquering these defects.

## Data Availability

The original contributions presented in the study are included in the article/Supplementary Material, further inquiries can be directed to the corresponding author.

## References

[B1] Abbott R. Albach D. Ansell S. Arntzen J. W. Baird S. J. E. Bierne N. (2013). Hybridization and Speciation. J. Evol. Biol. 26, 229–246. 10.1111/j.1420-9101.2012.02599.x 23323997

[B2] Agca C. A. Tuzcu M. Gencoglu H. Akdemir F. Ali S. Sahin K. (2012). Lycopene Counteracts the Hepatic Response to 7,12-dimethylbenz[a]anthracene by Altering the Expression of Bax, Bcl-2, Caspases, and Oxidative Stress Biomarkers. Pharm. Biol. 50, 1513–1518. 10.3109/13880209.2012.688057 22978712

[B3] Bartley D. M. Rana K. Immink A. J. (2001). The Use of Inter-specific Hybrids in Aquaculture and Fisheries. Rev. Fish. Biol. Fisher 10, 325–337. 10.1023/A:1016691725361

[B4] Barton N. H. Hewitt G. M. (1989). Adaptation, Speciation and Hybrid Zones. Nature 341, 497–503. 10.1038/341497a0 2677747

[B5] Birchler J. A. Yao H. Chudalayandi S. (2006). Unraveling the Genetic Basis of Hybrid Vigor. Proc. Natl. Acad. Sci. U.S.A. 103, 12957–12958. 10.1073/pnas.0605627103 16938847 PMC1559732

[B52] Braasch I. Postlethwait J. H. (2012). “Polyploidy in Fish and the Teleost Genome Duplication,” in Polyploidy and Genome Evolution (Springer), 341–383.

[B6] Campo D. Machado-Schiaffino G. Horreo J. L. Garcia-Vazquez E. (2009). Molecular Organization and Evolution of 5S rDNA in the Genus Merluccius and Their Phylogenetic Implications. J. Mol. Evol. 68, 208–216. 10.1007/s00239-009-9207-8 19247563

[B7] Chen J. Luo M. Li S. Tao M. Ye X. Duan W. (2018). A Comparative Study of Distant Hybridization in Plants and Animals. Sci. China Life Sci. 61, 285–309. 10.1007/s11427-017-9094-2 28861869

[B8] Chonghaile T. N. Sarosiek K. A. Vo T.-T. Ryan J. A. Tammareddi A. Moore V. D. G. (2011). Pretreatment Mitochondrial Priming Correlates with Clinical Response to Cytotoxic Chemotherapy. Science 334, 1129–1133. 10.1126/science.1206727 22033517 PMC3280949

[B9] Comber S. C. L. Smith C. (2004). Polyploidy in Fishes: Patterns and Processes. Biol. J. Linn. Soc. 82, 431–442. 10.1111/j.1095-8312.2004.00330.x

[B10] Coyne J. A. Orr H. A. (2004). Speciation. Sunderland, MA: Sinauer Associates.

[B11] Deng J. Shimamura T. Perera S. Carlson N. E. Cai D. Shapiro G. I. (2007). Proapoptotic BH3-Only BCL-2 Family Protein BIM Connects Death Signaling from Epidermal Growth Factor Receptor Inhibition to the Mitochondrion. Cancer Res. 67, 11867–11875. 10.1158/0008-5472.can-07-1961 18089817

[B12] Fuchs Y. Steller H. (2011). Programmed Cell Death in Animal Development and Disease. Cell 147, 742–758. 10.1016/j.cell.2011.10.033 22078876 PMC4511103

[B13] Galluzzi L. Vitale I. Aaronson S. A. Abrams J. M. Adam D. Agostinis P. (2018). Molecular Mechanisms of Cell Death: Recommendations of the Nomenclature Committee on Cell Death 2018. Cell Death Differ. 25, 486–541. 10.1038/s41418-017-0012-4 29362479 PMC5864239

[B14] He W. Qin Q. Liu S. Li T. Wang J. Xiao J. (2012). Organization and Variation Analysis of 5S rDNA in Different Ploidy-Level Hybrids of Red Crucian Carp × Topmouth Culter. PloS one 7, e38976. 10.1371/journal.pone.0038976 22720007 PMC3377697

[B15] He W. Xie L. Li T. Liu S. Xiao J. Hu J. (2013). The Formation of Diploid and Triploid Hybrids of Female Grass Carp × Male blunt Snout Bream and Their 5S rDNA Analysis. BMC Genet. 14, 110. 10.1186/1471-2156-14-110 24267392 PMC4222567

[B16] Hill W. G. (2013). “Selective Breeding,” in Brenner's Encyclopedia of Genetics. Second Edition (London: Academic Press), 1, 371–373. 10.1016/b978-0-12-374984-0.01390-5

[B17] Hu J. Liu S. Xiao J. Zhou Y. You C. He W. (2012). Characteristics of Diploid and Triploid Hybrids Derived from Female *Megalobrama amblycephala* Yih×male Xenocypris Davidi Bleeker. Aquaculture 364-365, 157–164. 10.1016/j.aquaculture.2012.08.025

[B53] Knytl M. Fornaini N. R. (2021). Measurement of Chromosomal Arms and FISH Reveal Complex Genome Architecture and Standardized Karyotype of Model Fish, Genus Carassius. Cells 10 (9), 2343. 10.3390/cells10092343 34571992 PMC8471844

[B18] Leggatt R. A. Iwama G. K. (2003). Occurrence of Polyploidy in the Fishes. Rev. Fish Biol. Fish. 13, 237–246. 10.1023/b:rfbf.0000033049.00668.fe

[B19] Liman N. Alan E. Bayram G. Gürbulak K. (2013). Expression of Survivin, Bcl-2 and Bax Proteins in the Domestic Cat (*Felis catus*) Endometrium during the Oestrus Cycle. Reprod. Domest. Anim. 48, 33–45. 10.1111/j.1439-0531.2012.02021.x 22471444

[B20] Liu S. Liu Y. Zhou G. Zhang X. Luo C. Feng H. (2001). The Formation of Tetraploid Stocks of Red Crucian Carp×common Carp Hybrids as an Effect of Interspecific Hybridization. Aquaculture 192 (2), 171–186. 10.1016/s0044-8486(00)00451-8

[B21] Liu S. Qin Q. Xiao J. Lu W. Shen J. Li W. (2007). The Formation of the Polyploid Hybrids from Different Subfamily Fish Crossings and its Evolutionary Significance. Genetics 176, 1023–1034. 10.1534/genetics.107.071373 17507678 PMC1894572

[B22] Liu S. (2010). Distant Hybridization Leads to Different Ploidy Fishes. Sci. China Life Sci. 53, 416–425. 10.1007/s11427-010-0057-9 20596907

[B23] Lucinda L. M. F. Aarestrup B. J. V. Peters V. M. de Paula Reis J. E. de Oliveira R. S. M. F. de Oliveira Guerra M. (2013). The Effect of the Ginkgo Biloba Extract in the Expression of Bax, Bcl-2 and Bone mineral Content of Wistar Rats with Glucocorticoid-Induced Osteoporosis. Phytother. Res. 27, 515–520. 10.1002/ptr.4747 22648569

[B24] Maheshwari S. Barbash D. A. (2011). The Genetics of Hybrid Incompatibilities. Annu. Rev. Genet. 45, 331–355. 10.1146/annurev-genet-110410-132514 21910629

[B25] Mallet J. (2005). Hybridization as an Invasion of the Genome. Trends Ecol. Evol. 20, 229–237. 10.1016/j.tree.2005.02.010 16701374

[B26] Mallet J. (2007). Hybrid Speciation. Nature 446, 279–283. 10.1038/nature05706 17361174

[B27] Martins C. Galetti P. M. Jr. (2001). Organization of 5S rDNA in Species of the Fish Leporinus: Two Different Genomic Locations Are Characterized by Distinct Nontranscribed Spacers. Génome 44, 903–910. 10.1139/gen-44-5-903 11681615

[B28] Muir W. M. Howard R. D. (1999). Possible Ecological Risks of Transgenic Organism Release when Transgenes Affect Mating success: Sexual Selection and the Trojan Gene Hypothesis. Proc. Natl. Acad. Sci. U.S.A. 96, 13853–13856. 10.1073/pnas.96.24.13853 10570162 PMC24154

[B29] Osabe K. Kawanabe T. Sasaki T. Ishikawa R. Okazaki K. Dennis E. S. (2012). Multiple Mechanisms and Challenges for the Application of Allopolyploidy in Plants. Int. J. Mol. Sci. 13, 8696–8721. 10.3390/ijms13078696 22942729 PMC3430260

[B30] Pasolini P. Costagliola D. Rocco L. Tinti F. (2006). Molecular Organization of 5S rDNAs in Rajidae (Chondrichthyes): Structural Features and Evolution of Piscine 5S rRNA Genes and Nontranscribed Intergenic Spacers. J. Mol. Evol. 62, 564–574. 10.1007/s00239-005-0118-z 16612546

[B31] Pawlowski W. P. Cande W. Z. (2005). Coordinating the Events of the Meiotic Prophase. Trends Cell Biol. 15, 674–681. 10.1016/j.tcb.2005.10.005 16257210

[B32] Pendas A. M. Moran P. Martinez J. L. Garcia-Vazquez E. (1995). Applications of 5S rDNA in Atlantic salmon, Brown trout, and in Atlantic salmon Brown trout Hybrid Identification. Mol. Ecol. 4, 275–276. 10.1111/j.1365-294x.1995.tb00220.x 7735532

[B33] Qin Q. He W. Liu S. Wang J. Xiao J. Liu Y. (2010). Analysis of 5S rDNA Organization and Variation in Polyploid Hybrids from Crosses of Different Fish Subfamilies. J. Exp. Zool. 314B, 403–411. 10.1002/jez.b.21346 20535772

[B34] Ramsey J. Schemske D. W. (1998). Pathways, Mechanisms, and Rates of Polyploid Formation in Flowering Plants. Annu. Rev. Ecol. Syst. 29, 467–501. 10.1146/annurev.ecolsys.29.1.467

[B35] Sajdak S. L. Reed K. M. Phillips R. B. (1998). Intraindividual and Interspecies Variation in the 5S rDNA of Coregonid Fish. J. Mol. Evol. 46, 680–688. 10.1007/pl00006348 9608050

[B36] Sarosiek K. A. Letai A. (2016). Directly Targeting the Mitochondrial Pathway of Apoptosis for Cancer Therapy Using BH3 Mimetics - Recent Successes, Current Challenges and Future Promise. Febs J. 283, 3523–3533. 10.1111/febs.13714 26996748 PMC5031512

[B37] Sarosiek K. A. Ni Chonghaile T. Letai A. (2013). Mitochondria: Gatekeepers of Response to Chemotherapy. Trends Cell Biol. 23, 612–619. 10.1016/j.tcb.2013.08.003 24060597 PMC3842421

[B38] Schumer M. Cui R. Powell D. L. Rosenthal G. G. Andolfatto P. (2016). Ancient Hybridization and Genomic Stabilization in a Swordtail Fish. Mol. Ecol. 25, 2661–2679. 10.1111/mec.13602 26937625

[B39] Singh R. Letai A. Sarosiek K. (2019). Regulation of Apoptosis in Health and Disease: the Balancing Act of BCL-2 Family Proteins. Nat. Rev. Mol. Cel Biol. 20, 175–193. 10.1038/s41580-018-0089-8 PMC7325303 30655609

[B40] Soltis P. S. Soltis D. E. (2009). The Role of Hybridization in Plant Speciation. Annu. Rev. Plant Biol. 60, 561–588. 10.1146/annurev.arplant.043008.092039 19575590

[B41] Song C. Liu S. Xiao J. He W. Zhou Y. Qin Q. (2012). Polyploid Organisms. Sci. China Life Sci. 55, 301–311. 10.1007/s11427-012-4310-2 22566086

[B42] Vaid N. Laitinen R. A. E. (2019). Diverse Paths to Hybrid Incompatibility in Arabidopsis. Plant J. 97, 199–213. 10.1111/tpj.14061 30098060

[B43] Venkatesh B. (2003). Evolution and Diversity of Fish Genomes. Curr. Opin. Genet. Develop. 13, 588–592. 10.1016/j.gde.2003.09.001 14638319

[B44] Wang J. Liu S. Xiao J. Tao M. Zhang C. Luo K. (2014). Evidence for the Evolutionary Origin of Goldfish Derived from the Distant Crossing of Red Crucian Carp × Common Carp. BMC Genet. 15, 33. 10.1186/1471-2156-15-33 24628745 PMC3995517

[B45] Wang J. Xiao J. Zeng M. Xu K. Tao M. Zhang C. (2015). Genomic Variation in the Hybrids of white Crucian Carp and Red Crucian Carp: Evidence from Ribosomal DNA. Sci. China Life Sci. 58, 590–601. 10.1007/s11427-015-4835-2 25804929

[B46] Wang S. Tang C. Tao M. Qin Q. Zhang C. Luo K. (2019). Establishment and Application of Distant Hybridization Technology in Fish. Sci. China Life Sci. 62, 22–45. 10.1007/s11427-018-9408-x 30554295

[B47] Wang Y. Yao J. Luo Y. Tan H. Huang X. Wang S. (2021). Two New Types of Homodiploid Fish and Polyploid Hybrids Derived from the Distant Hybridization of Female Koi Carp and Male Bighead Carp. Mar. Biotechnol. 23 (4), 628–640. 10.1007/s10126-021-10050-7 34401979

[B48] Wasko A. P. Martins C. Wright J. M. Galetti Jr. P. M. Jr. (2001). Molecular Organization of 5S rDNA in Fishes of the Genus Brycon. Génome 44, 893–902. 10.1139/gen-44-5-893 11681614

[B49] Wong T. T. Zohar Y. (2015). Production of Reproductively Sterile Fish by a Non-transgenic Gene Silencing Technology. Sci. Rep. 5, 15822. 10.1038/srep15822 26510515 PMC4625178

[B50] Zhao T. Tao X. Feng S. Wang L. Hong H. Ma W. (2018). LncRNAs in Polyploid Cotton Interspecific Hybrids Are Derived from Transposon Neofunctionalization. Genome Biol. 19, 195. 10.1186/s13059-018-1574-2 30419941 PMC6233382

[B51] Zohar Y. (1989). Endocrinology and Fish Farming: Aspects in Reproduction, Growth, and Smoltification. Fish. Physiol. Biochem. 7, 395–405. 10.1007/bf00004734 24221799

